# Intracranial Arterial Calcification and Its Impact on Subsequent Ischemic Cerebral Events: A Retrospective Cohort Study

**DOI:** 10.3390/diagnostics16183037

**Published:** 2026-09-19

**Authors:** Philipp Deisl, Stephanie Mangesius, Marie-Christine Pali, Maximilian Lutz, Michael Knoflach, Lukas Mayer-Suess, Stefan Kiechl, Gerlig Widmann, Astrid E. Grams, Elke R. Gizewski

**Affiliations:** 1 Department of Radiology, Medical University of Innsbruck, Anichstrasse 35, 6020 Innsbruck, Austria; philipp.deisl@i-med.ac.at (P.D.); marie-christine.pali@i-med.ac.at (M.-C.P.); maximilian.lutz@i-med.ac.at (M.L.); gerlig.widmann@i-med.ac.at (G.W.); astrid.grams@i-med.ac.at (A.E.G.); elke.gizewski@i-med.ac.at (E.R.G.); 2 Neuroimaging Research Core Facility, Medical University of Innsbruck, Anichstrasse 35, 6020 Innsbruck, Austria; 3 VASCage, Centre on Clinical Stroke Research, 6020 Innsbruck, Austria; michael.knoflach@i-med.ac.at (M.K.); stefan.kiechl@i-med.ac.at (S.K.); 4 Department of Neurology, Medical University of Innsbruck, 6020 Innsbruck, Austria; lukas.mayer@i-med.ac.at

**Keywords:** recurrent stroke, intracranial arterial calcification, atherosclerosis, CTA imaging, imaging biomarker

## Abstract

**Background**: Intracranial arterial calcification (IAC), commonly detected on CT/CTA during ischemic stroke work-up, may reflect atherosclerotic burden relevant to recurrence. We evaluated whether CTA-detected IAC was associated with subsequent ischemic cerebral events. **Methods**: This retrospective single-center cohort included patients with ischemic stroke or transient ischemic attack admitted to a tertiary stroke unit between 2010 and 2023. IAC was assessed on index-event CTA using volumetric quantification and the Babiarz score. Time to first recurrent ischemic cerebrovascular event was analyzed using multivariable Cox regression. Robustness was evaluated through adjustment for stroke etiology and CTA acquisition parameters, penalized regression, bootstrap internal validation, and competing-risk analysis accounting for death. **Results**: Among 556 patients (median age, 71.5 years [IQR, 62.3–78.6]; 354 male), 60 (10.8%) experienced recurrence during a median follow-up of 3.5 years (IQR, 1.8–7.5). Median IAC volume was higher in patients with recurrence than in those without recurrence (45.0 mm^3^ [IQR, 14.1–143.2] vs. 18.3 mm^3^ [IQR, 0–105.6]; *p* = 0.01). A one-unit increase in log_10_(IAC + 1), corresponding to a tenfold increase in IAC + 1, was associated with a higher recurrence hazard (HR, 1.63; 95% CI, 1.18–2.24; *p* = 0.003). The model had a C-index of 0.727, and the association remained consistent across sensitivity analyses. Babiarz scores correlated strongly with volumetric IAC measurements in the intracranial carotid and vertebral arteries (ρ ≥ 0.93; *p* < 0.01). **Conclusions**: Greater CTA-derived IAC burden was associated with a higher hazard of recurrent ischemic cerebrovascular events. Quantitative IAC assessment warrants further investigation as a potential imaging marker of recurrence risk, although external validation is required before clinical implementation.

## 1. Introduction

Stroke remains one of the most common causes of death and the largest contributor to disability-adjusted life-years globally [1]. The cost caused by stroke in Europe was €60 billion in 2017 alone [2]. The number of people living with stroke and its consequences is expected to increase in the next decades with an aging population [3]. Therefore, identifying reliable prognostic markers that allow early risk stratification is of critical importance to reduce cost and, more importantly, reduce the incidence of recurrent ischemic cerebral events (ICE) such as transient ischemic attack (TIA) or stroke.

Large artery atherosclerosis is one of the leading causes of stroke [4], and arterial calcifications are understood to act as a marker for atherosclerotic burden [5,6]. Intracranial arterial calcifications (IAC) are a common incidental finding on computed tomography (CT) scans performed as part of a routine diagnostic workup for stroke patients [7]. Due to their common presence and well-documented relevance of quantifying calcified plaques in cardiac risk stratification [8], IAC and its potential role in ICE has received increased attention.

Recent literature has shown a correlation between IAC and stroke [9], but data on the influence of IAC on the recurrence of either TIA or stroke are scarce. To address this knowledge gap, we systematically evaluated IAC on CTA using both quantitative volumetry and qualitative Babiarz scores for thickness and circumferential extent. By integrating these assessment methods in a retrospective cohort design, we aim to provide evidence on the relevance of CTA-quantified IAC for recurrence risk stratification in patients with IEC, thereby expanding current understanding of the contribution of intracranial atherosclerotic disease in recurrent cerebrovascular risk and assisting clinicians in identifying patients at risk.

The study was designed and reported in accordance with the STROBE (Strengthening the Reporting of Observational Studies in Epidemiology) guidelines for cohort studies [10].

## 2. Materials and Methods

### 2.1. Setting

**Figure 1:**
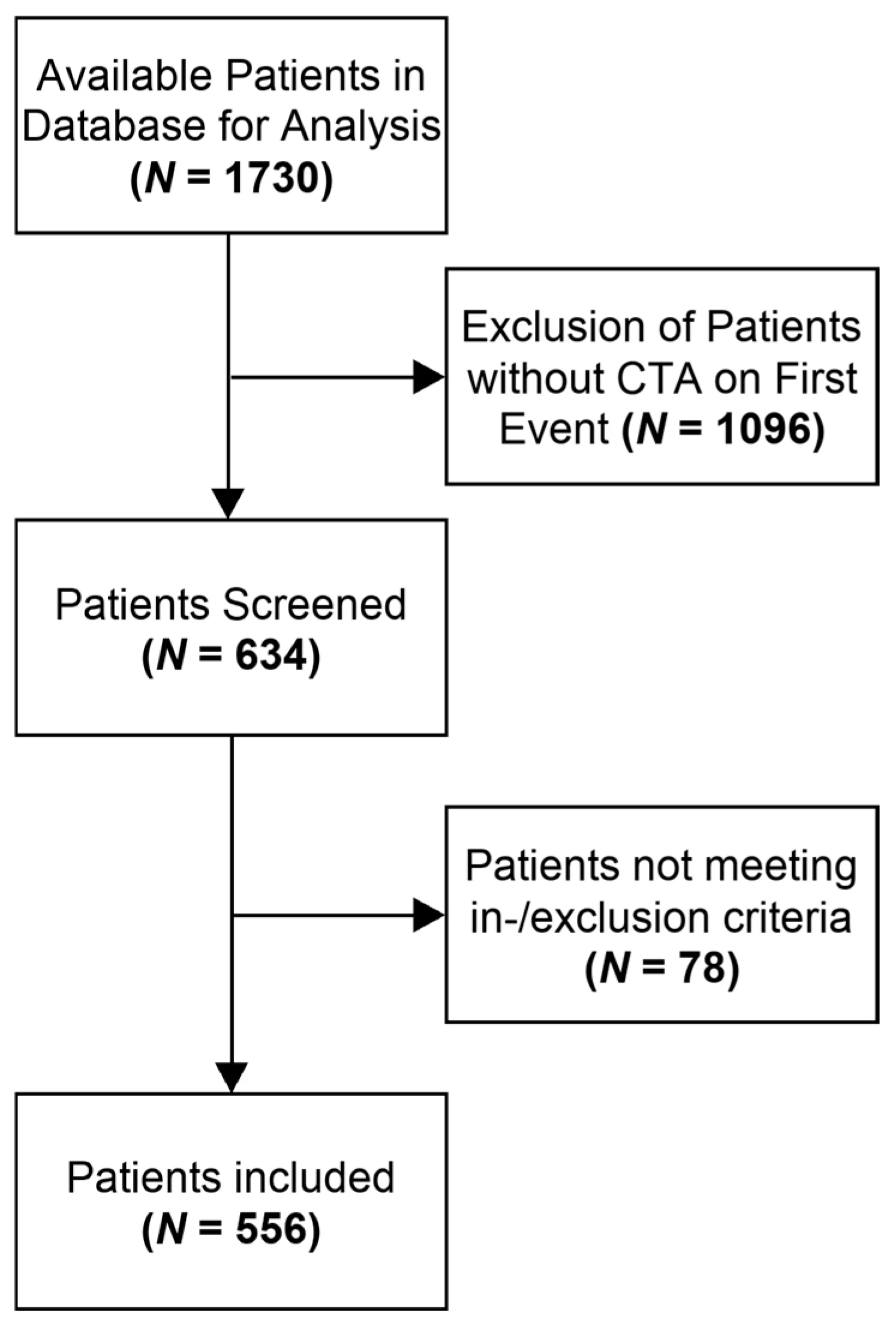
Flowchart illustrating the patient selection process. Downward arrows indicate progression to the next stage of patient selection. Rightward arrows indicate patients excluded at each stage.

We conducted a retrospective cohort study as part of the “Retrospective pilot project: Imaging Biomarkers for Vascular Diseases and Vascular Aging” including patients admitted to the local stroke unit of our tertiary academic center who experienced their first ICE between January 2010 and February 2023. As this study is part of a retrospective pilot project, the study size was determined by the number of eligible patients, who were identified through the stroke unit’s admission registry and were first screened for the availability of CTA at the index event. Only patients with available CTA were subsequently assessed against the remaining inclusion criteria (including technically evaluable IAC measurements), with detailed clinical and imaging variables abstracted specifically for this cohort. The objective was to assess the total IAC burden at the time of the first event and to determine whether IAC is independently associated with recurrent ICE. Inclusion criteria comprised being of legal age in Austria (over 18 years), being admitted with an acute ischemic stroke or TIA, having complete qualitative and quantitative IAC evaluation, and having available CTA imaging of the intracranial vessels during the first stroke/TIA dependent admission. TIA and ischemic stroke were classified as an ICE. Exclusion criteria comprised inadequate imaging quality and not meeting the inclusion criteria. IAC was analyzed on CTA studies of the index ICE. During follow-up, patients were grouped according to whether they had a recurrent ICE or only one ICE. According to these criteria, all patients documented in our database at the time of the data cutoff, 24 June 2025, were screened (Figure 1).

### 2.2. Variables and Data Collection

Data were collected and followed up retrospectively using records of the clinical information system as well as imaging data (CT, MRI, catheter-based digital subtraction angiography (DSA)) from 2010 onwards. Recurrent ischemic cerebrovascular events were ascertained by treating neurologists, including stroke specialists at a tertiary-care center with a dedicated stroke unit, based on clinical assessment and the available medical records. Neuroimaging findings were considered when available, but imaging confirmation of a corresponding ischemic lesion was not required for event ascertainment [11,12]. Clinical information from transfer reports, as well as imaging studies performed in external hospitals before the transfer to our local stroke unit, and hospital discharge letters from outside hospitals were also included in case they offered sufficient information.

### 2.3. Qualitative and Quantitative Assessment of IAC

**Figure 2:**
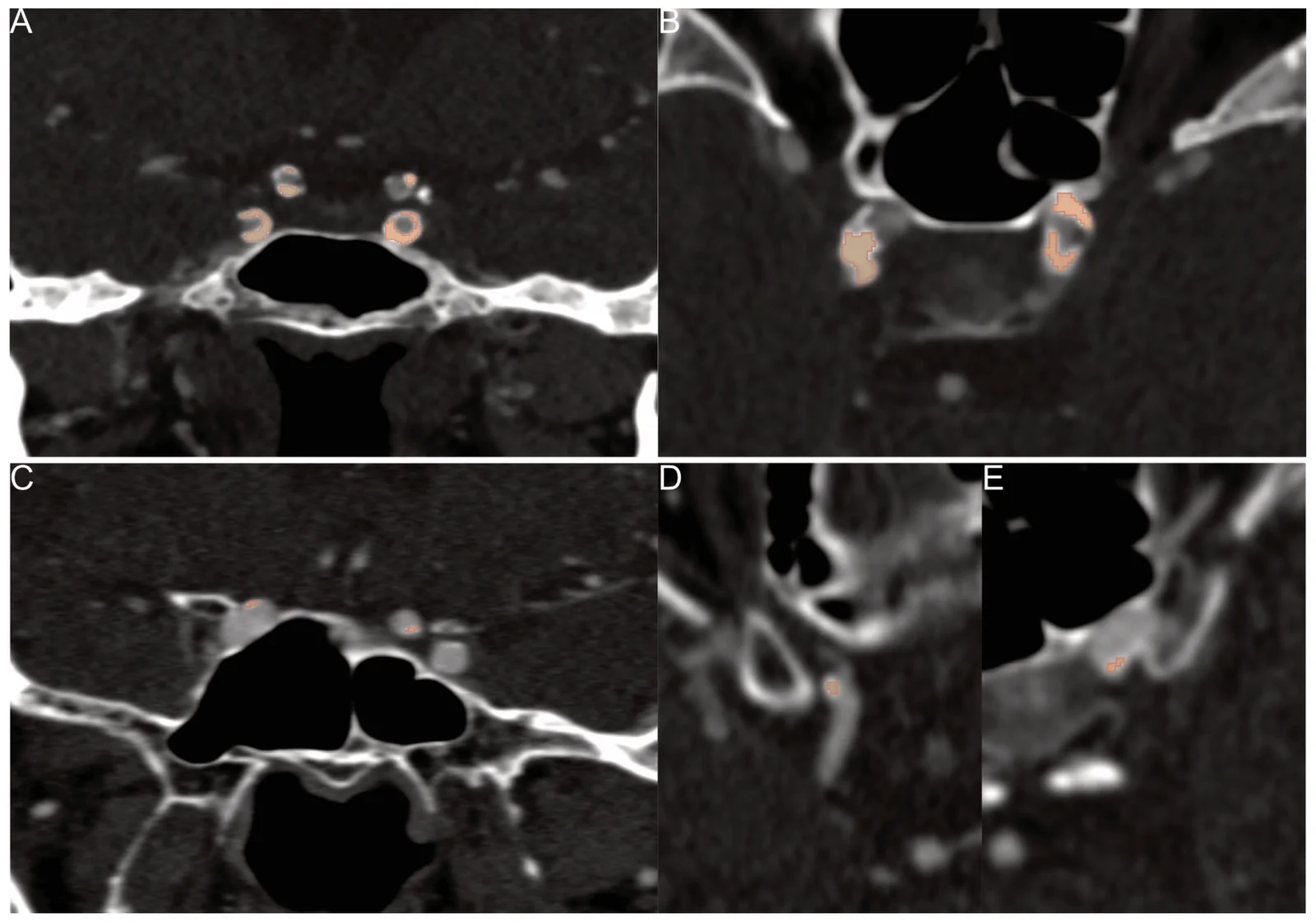
(**A**,**B**) Patient with recurrent ICE and IAC load corresponding to a Babiarz score of 4 (circumferential extent and thickness) in both ICAs; (**C**–**E**) Patient with no recurrence and IAC load corresponding to a Babiarz score of 2 (circumferential extent and thickness) in both ICAs. The orange overlays delineate the areas of intracranial arterial calcification segmented for volumetric assessment.

IAC was assessed using both intracranial and intra-/extracranial CTA scans (Figure 2). CTA scans were performed on SOMATOM Definition Flash (Siemens Healthineers, Forchheim, Germany; 312, 56%), SOMATOM Definition AS (Siemens Healthineers, Forchheim, Germany; 105, 19%), SOMATOM Drive (Siemens Healthineers, Forchheim, Germany; 33, 6%), GE Revolution HD (GE Medical Systems, LLC, Waukesha, WI, USA; 22, 4%), and SOMATOM Definition AS+ (Siemens Healthineers, Forchheim, Germany; 11, 2%). 13% (*n* = 73) were performed on other models, such as the GE Optima CT660 (GE Medical Systems, LLC, Waukesha, WI, USA), GE Revolution Apex (GE Medical Systems, LLC, Waukesha, WI, USA), or Philips Brilliance 64 (Philips Medical Systems Nederland B.V., Best, The Netherlands). Slice thickness ranged from 0.265 mm to 2.5 mm. Kilo voltage peak (KVP) ranged from 70 to 140 kV, and mAs values ranged from 39.6 to 460. The most administered contrast agent was Ultravist (Bayer Austria Ges.m.b.H., Vienna, Austria; 458, 82.4%), followed by Accupaque (GE Healthcare AS, Oslo, Norway; 26, 4.7%), and other agents, such as Jopamiro (Bracco Österreich GmbH, Vienna, Austria) or Unilux (Sanochemia Pharmazeutika GmbH, Neufeld an der Leitha, Austria; 4, 0.7%). No contrast agent was stated in 68 (12.2%) cases. Contrast agent volume ranged from 40 to 370 mL. Total IAC volume was evaluated on CTA images with a minimal cutoff of 600 HU to reliably distinguish contrast agent from calcification, which is in line with already published literature [13,14,15].

Evaluation of IAC was performed using 3D-Slicer Software (Version 5.6.1; RRID: SCR_005619; https://www.slicer.org/) [16,17]. First, IAC was segmented on the intracranial carotid artery starting from the C2 segment according to the classification of Bouthillier [18], as well as the M1-segment of the medial cerebral artery (MCA), the V4-segment of the vertebral artery, and the basilar artery (Figure 2). Subsequently, to minimize potential observer bias, the raters (P.D.; M.L.) were blinded to the outcome as well as clinical variables during the segmentation and evaluation process. IAC was qualitatively evaluated using the Babiarz score. In brief, the maximum circumferential extent (0 = none, 1 = dots of calcification, 2 ≤ 90°, 3 = 90–270°, 4 ≥ 270°) as well as plaque thickness (0 = none, 1 ≤ 1 mm, 2 ≤ 2 mm, 3 ≤ 3 mm, 4 ≤ 4 mm) were scored on a single image slice [19]. The final Babiarz score (0–4) was defined as the higher of the maximum circumferential extent score and the maximum plaque thickness score. Finally, quantitative IAC volumetry of the segmented parts in cubic millimeters [mm^3^] was performed by using 3D-Slicer software.

Intraobserver and interobserver reliability were assessed in a stratified random subset of 32 examinations from the final study cohort after a washout period of around 12 months. Patients were stratified according to total IAC burden into four groups: no detectable IAC, the lower, middle, and upper tertiles of total IAC volume. Using computer-generated random numbers with a predefined seed, eight examinations were randomly selected from each stratum to ensure coverage of the full range of calcification burden. The same examinations were used for both reliability analyses. Repeated measurements were performed using a blinded dataset that did not contain the original calcification measurements or clinical outcome variables.

### 2.4. Clinical Data

Cardiovascular risk factors evaluated in this study comprised binary-scaled cardiovascular risk factors such as hypertension, dyslipidemia, active smoking, type 1 or 2 diabetes mellitus, atrial fibrillation, as well as chronic renal insufficiency. Stroke etiology was classified according to the Trial of Org 10172 in Acute Stroke Treatment (TOAST) classification as large-artery atherosclerosis (LAA), small-vessel occlusion, cardio-embolism, other determined etiology, or undetermined etiology [20]. Demographic data comprised sex and age at the time of the initial ICE.

### 2.5. Statistical Analysis

Categorical variables were presented as numbers and percentages, continuous variables as mean (±standard deviation) or median (IQR). Due to the right-skewed distribution of the total calcification volumes and to handle values of 0, the data were transformed using the formula log_10_ (total IAC + 1). The age variable exhibited a non-linear relationship (Box-Tidwell *p* = 0.02) and was therefore modeled using both z-standardization and a quadratic transformation to account for the non-linear distribution in the Cox proportional hazards regression model.

For correlation of the ordinally scaled Babiarz score with the volumetric measurements, Spearman’s rank correlation was used. Group comparisons were performed using the *t*-test or Mann–Whitney U test for quantitatively scaled variables, depending on whether they are normally distributed. Cardiovascular risk factors (arterial hypertension, dyslipidemia, active smoking, diabetes mellitus type I/II, atrial fibrillation) were diagnosed by physicians based on discharge reports or defined by the intake of the respective medication (Statins, PCSK-9 inhibitors, Insulin, Metformin, etc.). Chronic renal insufficiency was defined as a persistent glomerular filtration rate below 60 mL/min for five days before and 30 days after the respective index ICE.

The primary analysis used Cox proportional hazards regression. The multivariable base model included age (transformed), sex, hypertension, diabetes mellitus, and atrial fibrillation. For our primary model log_10_(IAC + 1) was added. Given the limited number of recurrent events, covariate selection for the primary multivariable Cox model was deliberately parsimonious to reduce the risk of overfitting. Dyslipidemia and chronic kidney disease were not included in the primary model to limit model complexity relative to the 60 observed events. The proportional hazards assumption was assessed using Schoenfeld residual-based tests. Model discrimination was evaluated using the concordance index (C-index), and model fit was compared using partial Akaike’s information criterion (AIC). The incremental contribution of IAC was additionally assessed by comparing the model without IAC with the model containing log_10_(IAC + 1) using the likelihood-ratio test and difference in AIC. To assess whether the association between IAC volume and recurrent events deviated from linearity on the log_10_(IAC + 1) scale, a restricted cubic spline Cox model was fitted as a sensitivity analysis. The spline model was compared with the corresponding linear Cox model using a likelihood-ratio test evaluating whether the additional spline terms improved model fit.

Several sensitivity analyses were performed to assess the robustness of the primary findings. These included adjustment for stroke etiology, specifically for LAA, and for CTA scanner and acquisition parameters. Scanner model and reconstructed slice thickness were selected as acquisition-related covariates because of their plausible influence on IAC quantification and consistent availability across the study period. Other acquisition parameters, like kVp and mAs, were not included because of limited comparability across scanner generations and acquisition protocols and to avoid overparameterization given the limited number of recurrent events. Further analyses comprised penalized Cox regression analysis with a regularization parameter of 0.1, internal bootstrap validation of the primary Cox model, and competing-risk analysis considering death as a competing event. Calibration of the primary Cox proportional hazards model was assessed primarily at 3 years, with an additional calibration analysis performed at 5 years. For each time point, patients were divided into five approximately equal-sized groups according to their predicted recurrence risk, and the mean predicted risk was compared with the corresponding observed risk estimated using the Kaplan–Meier method within each group. The 45-degree line represented perfect calibration. As a secondary analysis, a multivariable logistic regression model was fitted using recurrent cerebrovascular event as a binary outcome. This analysis was explicitly considered a sensitivity analysis rather than the primary analysis, because it does not account for differences in follow-up duration or censoring. The logistic model included the same clinical covariates and log_10_(IAC + 1) as the primary Cox model. Odds ratios (ORs), 95% CIs, and *p*-values were reported. Discrimination of the logistic models was assessed using the area under the receiver operating characteristic curve (AUC). AUC was calculated for the clinical model without IAC and for the corresponding model including IAC. Bootstrap resampling was used to estimate the confidence interval for the difference in AUC. Analyses were performed using complete cases for the variables included in each respective model. No imputation of missing data was performed.

A two-sided *p*-value < 0.05 was considered statistically significant. Because the spline model did not demonstrate evidence of non-linearity, the linear log_10_(IAC + 1) specification was retained for the primary analysis. The assumption of a linear association between the ordinal Babiarz score and the log hazard was assessed by comparing models treating the score as a linear versus categorical term using a likelihood-ratio test as there was no evidence of departure from linearity for the Babiarz score (likelihood-ratio χ^2^ = 3.33, df = 3; *p* = 0.34).

Data manipulation and transformation were made using R statistical software (Version 4.3.3; R Foundation for Statistical Computing, Vienna, Austria) with the tidyverse, readxl, writexl, lubridate, car, haven, patchwork, pROC, broom, survival and ggpubr packages. Statistical analysis was performed using IBM SPSS Statistics (Version 30.0, IBM Corp., Armonk, NY, USA) as well as Python (Version 3.13; Python Software Foundation, Beaverton, OR, USA) including pandas, scipy, statsmodels, lifelines, and related packages. All analyses and figures were generated using a single reproducible analysis workflow.

### 2.6. Ethics

This study is part of the “Retrospective pilot project: Imaging Biomarkers for Vascular Diseases and Vascular Aging” and was approved by the Ethics Committee of the Medical University of Innsbruck (Approval number: 1429/2021; Date: 5 September 2022). The necessity of informed consent was waived due to the retrospective character of this project, using anonymized data and imaging.

### 2.7. Use of Generative AI

Large language models (GPT-5.2 Thinking, OpenAI, San Francisco, CA, USA) were used occasionally to assist with code generation for data processing and figure preparation and with minor language editing. All model-generated outputs were critically reviewed and verified by the authors before use.

## 3. Results

Of 634 patients with available CTA, 78 (12.3%) were excluded (Figure 1), most commonly because of luminal attenuation above the 600-HU threshold (*n* = 42, 53.8% of exclusions). Other reasons included insufficient image quality due to motion or artifacts (*n* = 13, 16.7%), inadequate technical imaging parameters or exportability (*n* = 13, 16.7%), incomplete vascular coverage or non-evaluable vessels (*n* = 8, 10.3%), and failure to meet clinical inclusion criteria (*n* = 2, 2.6%) (Supplementary Table S1).

Baseline characteristics are provided in Table 1. Median age was 71.5 years (IQR 62.3–78.6), and 63.7% were male. The index cerebrovascular event was an ischemic stroke in 534 patients (96.0%) and a TIA in 22 patients (4.0%). Among the 60 patients with recurrent events, 47 (78.3%) experienced a recurrent ischemic stroke and 13 (21.7%) experienced a recurrent TIA. The most prevalent stroke etiology was large artery atherosclerosis (182, 32.7%), followed by cardio-embolic stroke (156, 28.1%), unclear etiology (150, 27%), small-vessel occlusion (38, 6.8%), and other etiologies (30, 5.4%). The median follow-up period was 3.5 years (IQR 1.8–7.5) and did not statistically differ between the recurrence group (3.6 years (2.5–6.7)) and recurrence-free group (3.4 years (1.7–7.8); *p* = 0.44).

### 3.1. Intracranial Arterial Calcification and Recurrence of an Ischemic Cerebral Event

**Figure 3:**
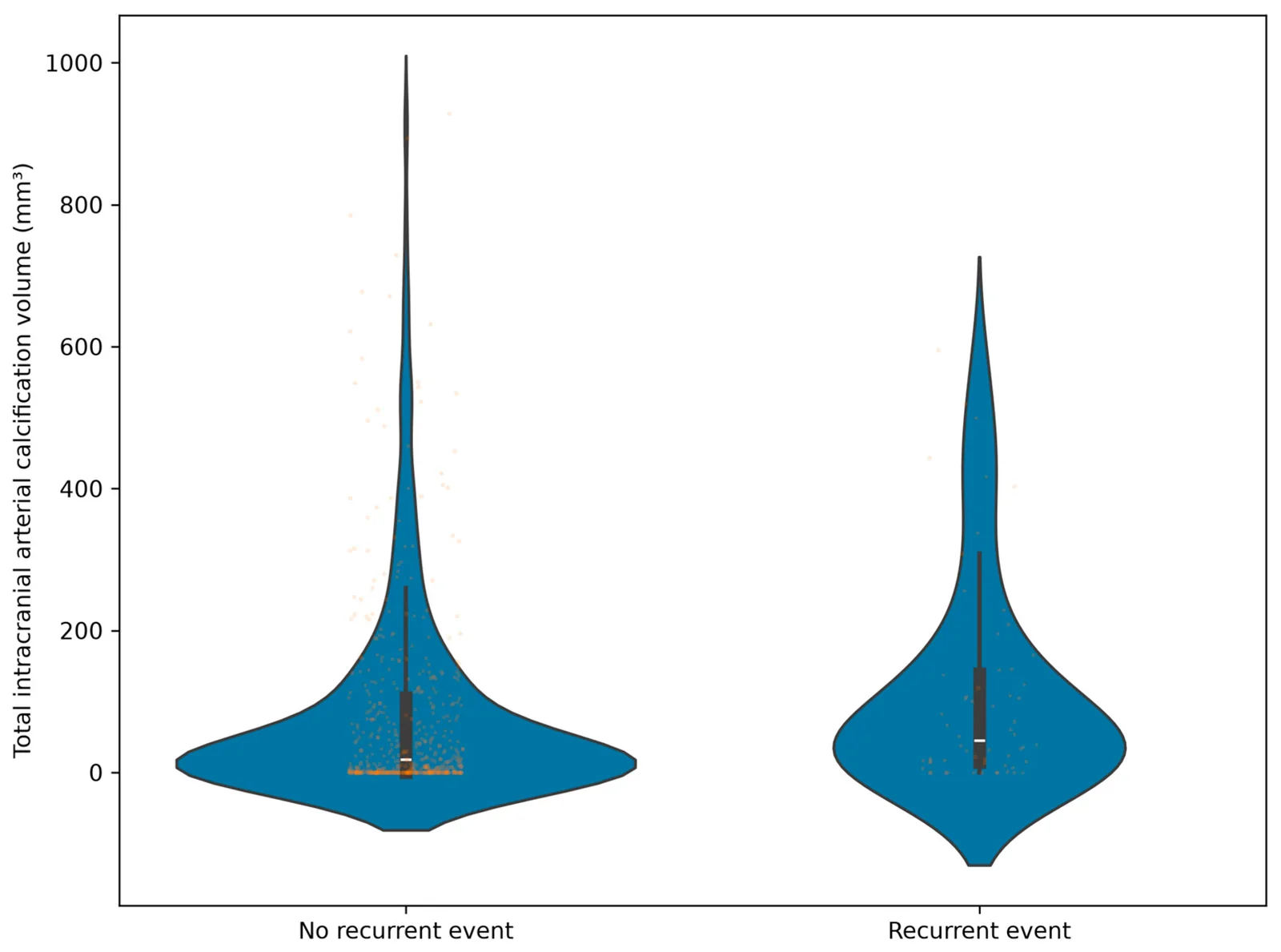
Violin plots and individual observations showing total intracranial arterial calcification (IAC) volume in patients without and with a recurrent event. Orange circles represent individual patient observations.

IAC was prevalent in 408 (73.4%) patients. Independent of their respective volume, IACs were most commonly found in the intracranial carotid artery (407; 73.2%), followed by the vertebral artery (95; 17.1%), basilar artery (5; 0.9%), and the MCA (2; 0.4%) (Table 2). Median total IAC volume in patients with a recurrent ICE was 45 mm^3^ (14.1–143.2), and for patients with no recurrent ICE was 18.3 mm^3^ (0–105.6), and differed significantly between the two groups (*p* = 0.01; Figure 3).

#### 3.1.1. Primary Time-to-Event Analysis

**Figure 4:**
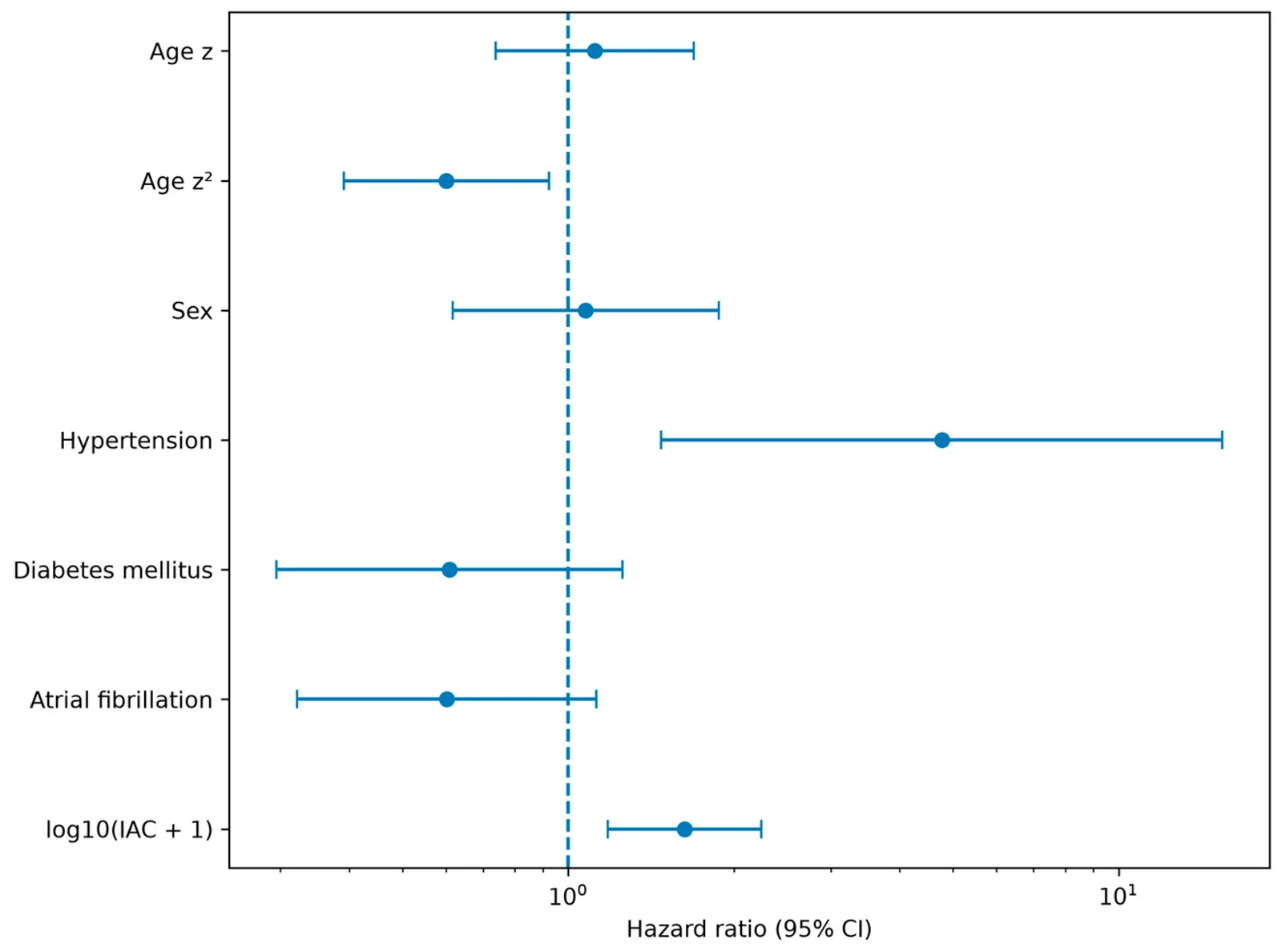
Forest Plot of the primary Cox model.

In the primary multivariable Cox proportional hazards model, higher IAC volume was significantly associated with an increased hazard of recurrent cerebrovascular events. A one-unit increase in log_10_(IAC + 1), corresponding to a tenfold increase in IAC + 1, was associated with a 63% higher hazard of recurrence (HR, 1.627; 95% CI, 1.180–2.243; *p* = 0.003) after adjustment for age, sex, hypertension, diabetes mellitus, and atrial fibrillation. The analysis included 554 patients, of whom 60 experienced a recurrent event (Table 3 and Figure 4).

Addition of IAC to the multivariable Cox model improved model fit, with a reduction in partial AIC from 617.16 in the corresponding base model containing the clinical covariates but not IAC to 609.97 in the primary model (ΔAIC = 7.19; likelihood-ratio test, χ^2^ = 9.2; *p* = 0.0024). The primary model showed an apparent C-index of 0.727 (95% bootstrap CI, 0.688–0.800), supporting an incremental prognostic contribution of IAC beyond the clinical covariates included in the base model. Internal validation using bootstrap resampling indicated modest optimism in model discrimination, with a mean optimism of 0.033 and an optimism-corrected C-index of 0.695 (Supplementary Figure S1). The proportional hazards assumption was adequately met, with Schoenfeld residual-based tests showing no evidence of violation for any covariate in the primary model (*p* = 0.11–0.879). Restricted cubic spline analysis provided no evidence of nonlinearity in the association between log_10_(IAC + 1) and recurrent cerebrovascular events (likelihood-ratio test, χ^2^ = 0.187; df = 4; *p* = 0.996). The spline model did not materially improve discrimination (C-index, 0.728 vs. 0.727 for the corresponding linear model) and showed a higher partial AIC (617.79 vs. 609.97), supporting the use of log_10_(IAC + 1) as a linear continuous exposure.

#### 3.1.2. Post Hoc Sensitivity Analyses

**Figure 5:**
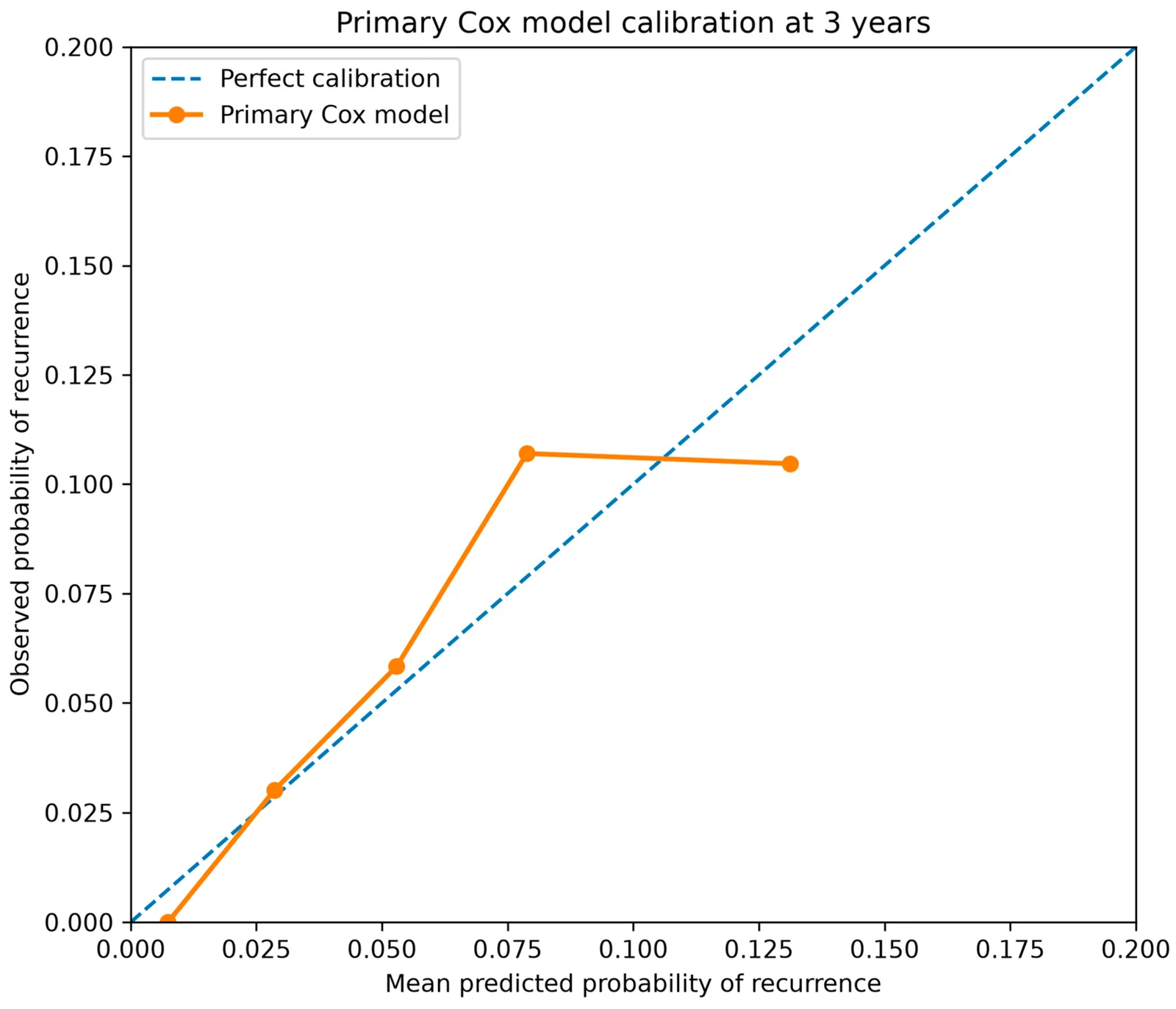
Calibration of the primary Cox proportional hazards model at 3 years. Predicted probabilities of recurrent cerebral events were compared with Kaplan–Meier-estimated observed probabilities across five approximately equal-sized groups according to predicted risk. The diagonal line represents perfect calibration.

The association between IAC and recurrent cerebrovascular events was robust across sensitivity analyses. Results also remained consistent after adjustment for stroke etiology (HR, 1.59; 95% CI, 1.13–2.24; *p* = 0.008), LAA (HR, 1.60; 95% CI, 1.14–2.24; *p* = 0.007), and CTA acquisition parameters for slice thickness and scan model (HR, 1.60; 95% CI, 1.14–2.24; *p* = 0.007), as well as in penalized Cox regression (HR, 1.28; 95% CI, 1.04–1.57; *p* = 0.019) and competing-risk analysis accounting for death (csHR, 1.78; 95% CI, 1.24–2.55; *p* = 0.002). Bootstrap validation yielded an optimism-corrected C-index of 0.695 (apparent C-index, 0.7275). The primary Cox model showed good agreement between predicted and observed risks of recurrent cerebral events at 3 years (Figure 5). Across five risk groups, the mean absolute difference between predicted and Kaplan–Meier-estimated risks was 0.0138. Calibration remained similar at 5 years, with a mean absolute difference of 0.0155 (Supplementary Figure S2). Secondary logistic regression showed a consistent association (OR, 1.42 95% CI, 1.004–2; *p* = 0.047). The base model without IAC yielded an AUC of 0.686, whereas addition of IAC increased the AUC to 0.701, although the incremental increase in AUC was modest and its bootstrap confidence interval included zero (−0.018–0.049) (Table 4).

**Figure 6:**
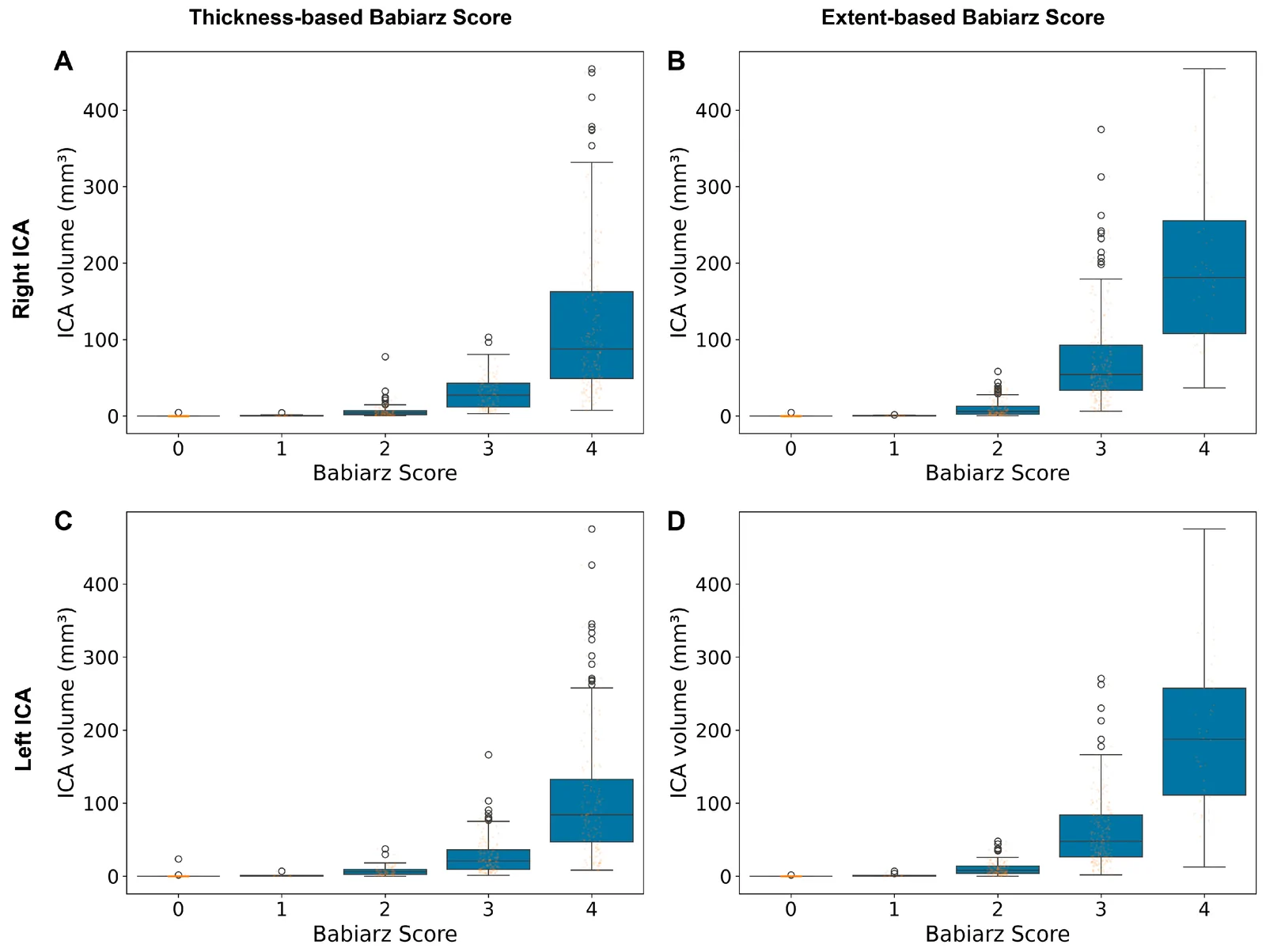
Correlation of the Babiarz score with the volumetric measurement for intracranial Internal Carotid Arteries. (**A**) Right-sided IAC volume by thickness-based score; (**B**) right-sided IAC volume by extent-based score; (**C**) left-sided IAC volume by thickness-based score; (**D**) left-sided IAC volume by extent-based score. Orange dots represent individual observations. White circles indicate outliers, defined as observations outside the boxplot whiskers.

**Figure 7:**
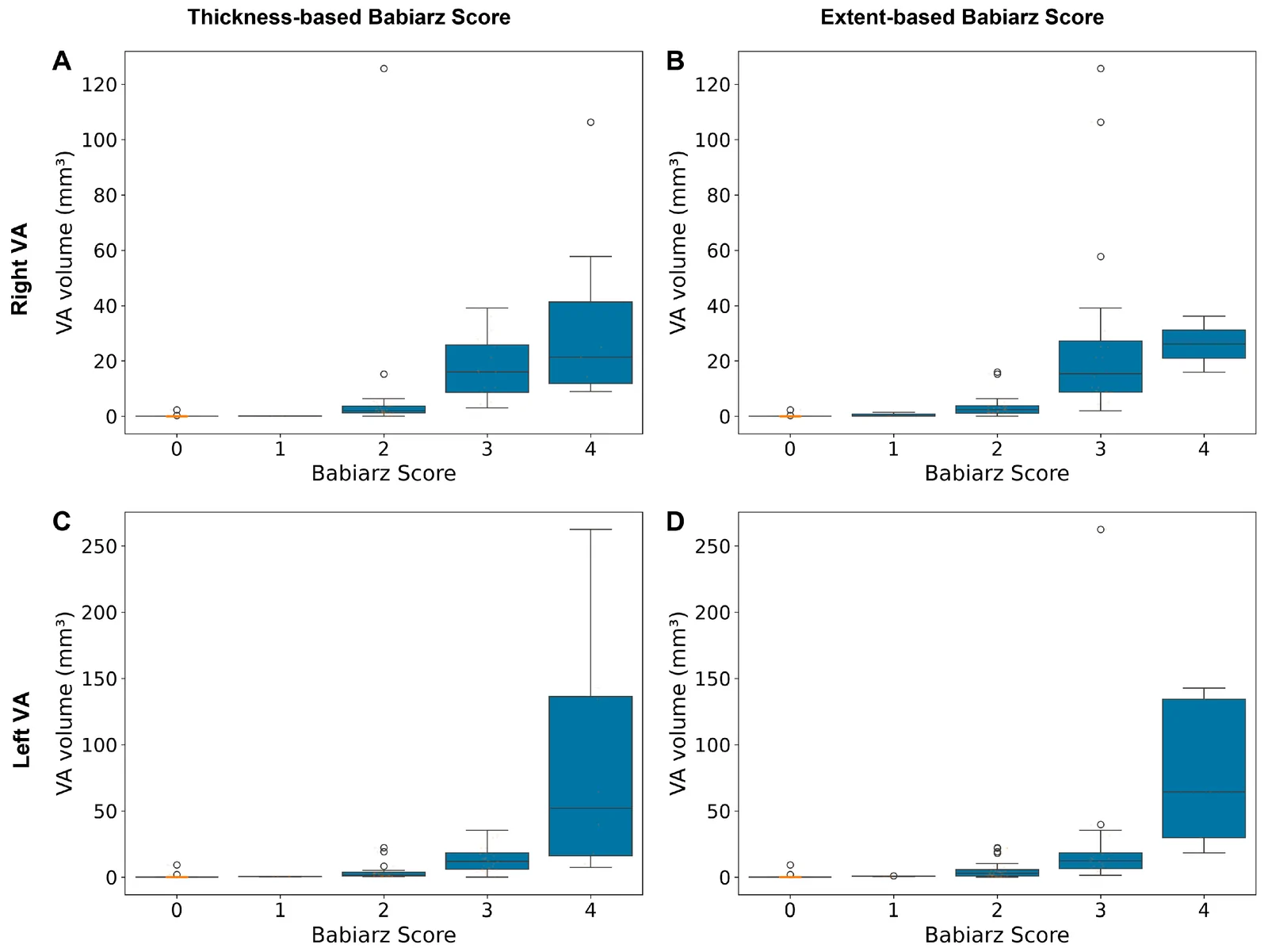
Correlation of the Babiarz score with the volumetric measurement for intracranial Vertebral Arteries. (**A**) Right-sided VA volume by thickness-based score; (**B**) right-sided VA volume by extent-based score; (**C**) left-sided VA volume by thickness-based score; (**D**) left-sided VA volume by extent-based score. Orange dots represent individual observations. White circles indicate outliers, defined as observations outside the boxplot whiskers.

Due to the small number of calcifications of the basilar artery and MCA, only the intracranial carotid artery and vertebral artery were assessed for correlation. Both Babiarz scores for Extent and Thickness showed excellent correlation with the volumetric measurement (Figure 6 and Figure 7). Results were also consistent when IAC burden was assessed using the ordinal Babiarz score, with each one-category increase associated with a higher hazard of recurrent cerebrovascular events (HR, 1.23; 95% CI, 1.01–1.50; *p* = 0.035).

#### 3.1.3. Intra- and Interobserver Reliability

Inter- and intraobserver reliability was evaluated in a stratified random subset of 32 examinations covering the full range of IAC burden. Quantitative calcification volume demonstrated excellent reliability. The absolute-agreement, single-measure intraobserver ICC was 0.9999 (95% CI, 0.9998–0.9999), and the corresponding interobserver ICC was 0.9993 (95% CI, 0.9986–0.9997). Bland–Altman analysis demonstrated negligible systematic bias. The mean intraobserver difference was −0.04 mm^3^ (95% CI, −0.95 to 0.86 mm^3^), with 95% limits of agreement from −4.96 to 4.88 mm^3^. The mean interobserver difference was −0.35 mm^3^ (95% CI, −2.53 to 1.84 mm^3^), with 95% limits of agreement from −12.21 to 11.52 mm^3^. There was no evidence of proportional bias in either the intraobserver (*p* = 0.947) or interobserver analysis (*p* = 0.456). Agreement for the maximum Babiarz score was excellent within the same observer (linearly weighted κ = 0.963; 95% CI, 0.912–1.000; *p* < 0.001) and substantial between observers (linearly weighted κ = 0.689; 95% CI, 0.546–0.833; *p* < 0.001).

## 4. Discussion

In this study of consecutive ischemic stroke or TIA patients admitted to the stroke unit of our tertiary academic center, those who experienced a recurrent ICE showed a higher total IAC volume at baseline CTA than those without recurrence. The observed association was consistent across sensitivity analyses. Additional adjustment for stroke etiology and specifically for large-artery atherosclerosis (LAA) resulted in similar effect estimates, with IAC remaining significantly associated with recurrence. These findings suggest that the observed association is not fully explained by LAA alone, although residual confounding by stroke etiology and other vascular characteristics cannot be excluded. Similarly, adjustment for available CTA acquisition parameters did not materially alter the association. Penalized Cox regression yielded a directionally consistent result, reducing concerns that the primary finding was driven solely by overfitting in the conventional multivariable model. Furthermore, competing-risk analysis accounting for death demonstrated a consistent association between IAC and recurrence (csHR, 1.78; 95% CI, 1.24–2.55; *p* = 0.002). Internal bootstrap validation resulted in an optimism-corrected C-index of 0.695 compared with an apparent C-index of 0.727, indicating moderate discrimination after correction for model optimism. The primary Cox model demonstrated generally acceptable calibration at 3 years, with similar findings at 5 years, although some deviations between predicted and observed recurrence risks were evident in the higher-risk groups. Secondary logistic regression was also directionally consistent with the primary time-to-event analysis, although the incremental improvement in AUC after adding IAC was modest and statistically uncertain. Thus, while IAC appears to provide information beyond the clinical covariates included in our models, the present findings do not support its use as a stand-alone clinical prediction tool.

Considering qualitative grading of a quantitative volumetric measure, the Babiarz scores showed excellent correlation with the volumetric measurement of IAC on CTA. Interestingly, this was even slightly better on posterior circulation vessels rather than anterior circulation measurements, for which the score was originally not developed. Although the Babiarz score derived from CTA appears to correlate more strongly in the V4 segment of the vertebral artery than in the intracranial carotid artery, a definitive physiological or anatomical rationale for this discrepancy has yet to be identified. Currently, there is no evidence or previous literature to investigate or explain this segment-dependent difference. Importantly, when the maximum Babiarz score was used as an alternative measure of IAC burden in the Cox model, each one-category increase was associated with a higher hazard of recurrent ICE (HR, 1.23; 95% CI, 1.01–1.50; *p* = 0.035).

To our knowledge, this is the first study to quantitatively assess IAC on CTA and to correlate the qualitative Babiarz score with CTA-based volumetry and recurrent ischemic events in a stroke/TIA cohort. Previous studies linking IAC to recurrent cerebrovascular events have relied exclusively on non-contrast CT, in which CTA-based volumetric analyses were lacking [15,21,22,23]. Clinical reasoning might suggest that initial stroke etiology pre-defines those at highest risk of stroke recurrence, rendering IAC measurement solely useful in those with large-artery atherosclerosis or small-vessel occlusion. A recent meta-analysis by Conte et al. reported increased odds of stroke incidence or recurrence in patients with IAC (OR, 1.31; 95% CI, 1.17–1.46) [9]. Similarly, a meta-analysis by Li et al., pooling data from 12 studies (*n* = 9346), reported that IAC was independently associated with both stroke occurrence (adjusted OR, 1.62; 95% CI, 1.18–2.23) and stroke recurrence (adjusted OR, 1.77; 95% CI, 1.25–2.51), although no significant association with post-stroke mortality was found [24]. Despite differences in study design and outcome definitions (incidence and recurrence), the available evidence collectively supports further investigation of IAC as a potential marker of subsequent cerebrovascular risk.

Strengths of this study include IAC characterization using both quantitative volumetric and qualitative measurement methods assessing circumferential extent and thickness. Our protocol and qualitative scoring algorithm were designed to assess IAC as objectively as possible and to provide initial evidence on the applicability of the Babiarz scoring system to CTA examinations and its correlation with CTA-based volumetric measurements, for which limited prior evidence exists. Quantitative IAC measurements demonstrated excellent intra- and interobserver reliability, with negligible systematic bias on Bland–Altman analysis, while agreement for the maximum Babiarz score was excellent within observers and substantial between observers. IAC assessment was performed blinded to the primary outcome and clinical variables, and qualitative assessments preceded volumetric analysis to minimize potential observer bias. Further strengths include the use of time-to-event analysis accounting for variable follow-up and complementary sensitivity analyses addressing model specification, stroke etiology, technical acquisition parameters, overfitting, and competing mortality. Despite potential challenges related to contrast administration [25], CTA is faster, more accessible, and easier to perform than other neurovascular imaging techniques (DSA, MRI), making it a suitable tool for the assessment of intracranial arterial pathology in acute and routine clinical settings. Aside from IAC, CTA images enable further measurements, such as intraplaque hemorrhage, degree of stenosis, or segmentation of the whole vessel [21,26,27].

Several limitations should be considered. First, the retrospective single-center design entails inherent risks of selection, information, and residual confounding. Inclusion was restricted to patients with available CTA at the index event and technically evaluable IAC measurements, which may have resulted in a non-representative study population and differential representation of stroke subtypes. Patients with small-vessel disease may be particularly underrepresented because CTA is not routinely obtained in all such patients, consistent with prior reports linking CTA utilization to stroke severity and suspected large-vessel pathology [28]. As these excluded patients were not part of our structured database, a direct comparison with the included cohort was not feasible. In addition, 78 patients with available CTA were excluded, predominantly because luminal attenuation was at, near, or above the predefined 600-HU threshold or because of insufficient image quality or technical limitations. Although this threshold was required to distinguish vascular contrast enhancement from calcification using the applied segmentation approach, these exclusions may have introduced additional selection bias. Incomplete clinical documentation inherent to retrospective data collection may have further contributed to complete-case and information bias.

Second, ascertainment of recurrent events depended on available clinical records, imaging, transfer reports, and external documentation. Although structured clinical follow-up by our colleagues from the Department of Neurology may have reduced the risk of incomplete outcome ascertainment [29], events occurring outside the regional healthcare system may have remained undocumented, particularly among patients who subsequently moved or were only temporarily residing in the region. Sociodemographic information that could have facilitated assessment of this potential loss to follow-up was unavailable because of the retrospective study design and local regulatory constraints. Although the time-to-event approach accounts for differing observed follow-up durations through censoring, incomplete ascertainment of events after loss to follow-up cannot be excluded.

Third, only 60 recurrent events occurred. We therefore deliberately used a parsimonious primary model to reduce the risk of overfitting. The consistency of the results in penalized Cox regression and bootstrap internal validation is reassuring but does not substitute for external validation. Moreover, the moderate optimism-corrected discrimination indicates that the current model should not be interpreted as a clinically validated prediction model. Finally, scanner and acquisition characteristics varied over the long study period. Although adjustment for available acquisition parameters yielded similar results, residual effects of technical heterogeneity cannot be completely excluded.

Quantitative segmentation of intracranial arterial calcification remains time-consuming and is currently not practical for routine clinical implementation. Automated approaches analogous to established coronary artery calcium quantification may therefore be required before volumetric IAC assessment can be incorporated into clinical workflows. Intracranial arteries pose particular technical challenges because of their close anatomical relationship to the skull base and other osseous structures, complicating automated differentiation of calcification from adjacent bone. Advances in automated image analysis and artificial intelligence may facilitate robust vessel segmentation [26] and calcium quantification [30], while substantially reducing the need for manual processing. Future prospective multicenter studies using standardized acquisition and automated IAC quantification are warranted to externally validate the present findings, establish clinically meaningful risk estimates, and determine whether IAC provides incremental value beyond established clinical and imaging markers for recurrent cerebrovascular risk stratification.

## 5. Conclusions

This study provides additional evidence that greater IAC burden, quantified volumetrically on CTA, is associated with a higher hazard of recurrent ICE in patients with stroke or TIA, complementing existing meta-analytic evidence linking IAC to stroke recurrence based predominantly on non-contrast CT and dichotomous assessment [9,24]. Therefore, the development of AI-based quantitative segmentation for automated volumetric assessment of intracranial arterial calcification may facilitate future clinical implementation of this imaging marker. Prospective multicenter studies are warranted to externally validate these findings and to determine whether IAC provides incremental value for recurrent cerebrovascular risk stratification.

## Figures and Tables

**Table 1:** Baseline characteristics of the study population.

	No Recurrent Event	Recurrent Event	Total
Age (years) Median (IQR)	71.5 (61.6–78.7)	72.6 (65.9–77.5)	71.5 (62.3–78.6)
Sex (male) *n* (%)	314 (63.3)	40 (66.7)	354 (63.7)
Hypertension *n* (%)	392 (79)	57 (95)	449 (80.8)
Dyslipidemia *n* (%)	402 (81)	54 (90)	456 (82)
Active Smoking *n* (%)	114 (23)	15 (25)	129 (23.2)
Diabetes Mellitus Type I/II *n* (%)	99 (20)	9 (15)	108 (19.4)
Atrial Fibrillation *n* (%)	148 (29.8)	14 (23.3)	162 (29.1)
Glomerular Filtration Rate < 60 *n* (%)	62 (12.5)	11 (18.3)	73 (13.1)
Oral anticoagulant after event *n* (%)	157 (31.7)	19 (31.7)	176 (31.7)
Antiplatelet therapy after event *n* (%)	314 (63.3)	47 (78.3)	361 (64.9)
Oral anticoagulant before event *n* (%)	67 (13.5)	9 (15)	76 (13.7)
Antiplatelet therapy before event *n* (%)	131 (26.4)	28 (46.7)	159 (28.6)
Etiology			
Large Artery Atherosclerosis *n* (%)	156 (31.5)	26 (43.3)	182 (32.7)
Cardio-embolic *n* (%)	143 (28.8)	13 (21.7)	156 (28.1)
Unclear *n* (%)	135 (27.2)	15 (25)	150 (27)
Microangiopathic *n* (%)	35 (7.1)	3 (5)	38 (6.8)
Other *n* (%)	28 (5.6)	2 (3.3)	30 (5.4)
IAC Volume			
Mean ± SD [mm^3^]	81.96 ± 140.41	111.21 ± 148.08	85.11 ± 141.42
Median [mm^3^]	18.33	44.97	21.74
Q1–Q3 [mm^3^]	0–105.57	14.1–143.17	0–111.86

**Table 2:** Distribution of IAC in different vessels.

	No Recurrent Event	Recurrent Event	Total
ICA right *n* (%)	330 (66.5)	47 (78.3)	377 (67.8)
ICA left *n* (%)	324 (65.3)	49 (81.7)	373 (67.1)
MCA (M1) right *n* (%)	0 (0)	0 (0)	0 (0)
MCA (M1) left *n* (%)	1 (0.2)	1 (1.7)	2 (0.4)
VA (V4) right *n* (%)	52 (10.5)	5 (8.3)	57 (10.3)
VA (V4) left *n* (%)	59 (11.9)	9 (15)	68 (12.2)
BA *n* (%)	5 (1)	0 (0)	5 (0.9)
Any IAC *n* (%)	358 (72.2)	50 (83.3)	408 (73.4)
Total *n* (%)	496 (100)	60 (100)	556 (100)

**Table 3:** Primary Cox proportional hazards regression analysis for recurrent cerebrovascular events.

Covariate	HR (95% CI)	*p*-Value
Age-standardized linear term	1.12 (0.74–1.69)	0.6
Age-standardized quadratic term	0.60 (0.39–0.92)	0.02
Sex (female vs. male)	1.08 (0.62–1.88)	0.797
Arterial Hypertension	4.77 (1.48–15.43)	0.009
Diabetes mellitus	0.61 (0.29–1.26)	0.179
Atrial fibrillation	0.6 (0.32–1.12)	0.111
log_10_(IAC volume + 1)	1.63 (1.18–2.24)	0.003

**Table 4:** Sensitivity analyses of the association between IAC and recurrent cerebrovascular events.

Analysis	Effect Estimate (95% CI)	*p*-Value
Primary Cox model	HR 1.63 (1.18–2.24)	0.003
Stroke etiology-adjusted	HR 1.59 (1.13–2.24)	0.008
LAA-adjusted	HR 1.60 (1.14–2.24)	0.007
CTA acquisition-adjusted	HR 1.60 (1.14–2.24)	0.007
Penalized Cox regression	HR 1.28 (1.04–1.57)	0.019
Competing-risk analysis	csHR 1.78 (1.24–2.55)	0.002
Babiarz score model *	HR * 1.23 (1.01–1.50)	0.035
Secondary logistic regression	OR 1.42 (1.00–2.00)	0.047

* For the Babiarz score model, the effect estimate represents the HR per one-category increase in the maximum Babiarz score; for all other Cox models, HRs refer to a one-unit increase in log_10_(IAC + 1).

## Data Availability

The data presented in this study are available upon request from the corresponding author due to ethical reasons.

## Supplementary Materials

The following supporting information can be downloaded at https://www.mdpi.com/article/10.3390/diagnostics16183037/s1, Table S1: Reasons for exclusion; Figure S1: Bootstrap internal validation of the multivariable Cox proportional hazards model for recurrent ischemic cerebral events; Figure S2: Calibration of the primary Cox proportional hazards model at 5 years. Predicted probabilities of recurrent cerebral events were compared with Kaplan–Meier-estimated observed probabilities across five approximately equal-sized groups according to predicted risk. The diagonal line represents perfect calibration.

## Abbreviations

The following abbreviations are used in this manuscript:

AUC	Area under the Curve
BA	Basilar Artery
CT	Computed Tomography
CTA	Computed Tomography Angiography
DSA	Digital Subtraction Angiography
HR	Hazard Ratio
HU	Hounsfield Units
IAC	Intracranial Arterial Calcification
ICA	Internal Carotid Artery
ICE	Ischemic Cerebral Events
IQR	Interquartile Range
IRB	Institutional Review Board
kVp	Peak tube voltage
LAA	Large Artery Atherosclerosis
mAs	Tube current-time product
MCA	Medial Cerebral Artery
MRI	Magnetic Resonance Tomography
OAC	Oral Anticoagulation
OR	Odds Ratio
SD	Standard Deviation
STROBE	Strengthening the Reporting of Observational Studies in Epidemiology
TFI	Thrombocyte Function Inhibitor
TIA	Transient Ischemic Attack
VA	Vertebral Artery
