# Supplementary material for: Intracranial Arterial Calcification and Its Impact on Subsequent Ischemic Cerebral Events: A Retrospective Cohort Study
Source: Diagnostics (Basel). 2026 Sep 19;16(18):3037. doi: 10.3390/diagnostics16183037 (PMC13605079; doi:10.3390/diagnostics16183037)
Supplement: Supplementary file 1 [file diagnostics-16-03037-s001.zip › diagnostics-4472440-supplementary.pdf]

Supplemental material

Supplementary Table S1 Reasons for exclusion

Primary reason for exclusion	*n* (%) [1]	% of CTA cohort [2]
Luminal attenuation at, near, or above the predefined 600-HU threshold	42 (53.8)	6.6
Motion artifacts or insufficient overall image quality	13 (16.7)	2.1
Inadequate slice thickness, reconstruction, image geometry, or image exportability	13 (16.7)	2.1
Incomplete vascular coverage or anatomically/technically non-evaluable vessels	8 (10.3)	1.3
Clinical inclusion criteria not met	2 (2.6)	0.3
Total	78 (100.0)	12.3

[1] Percentage of all excluded patients with available CTA (*n*=78).

[2] Percentage of all patients with available CTA (*n*=634)

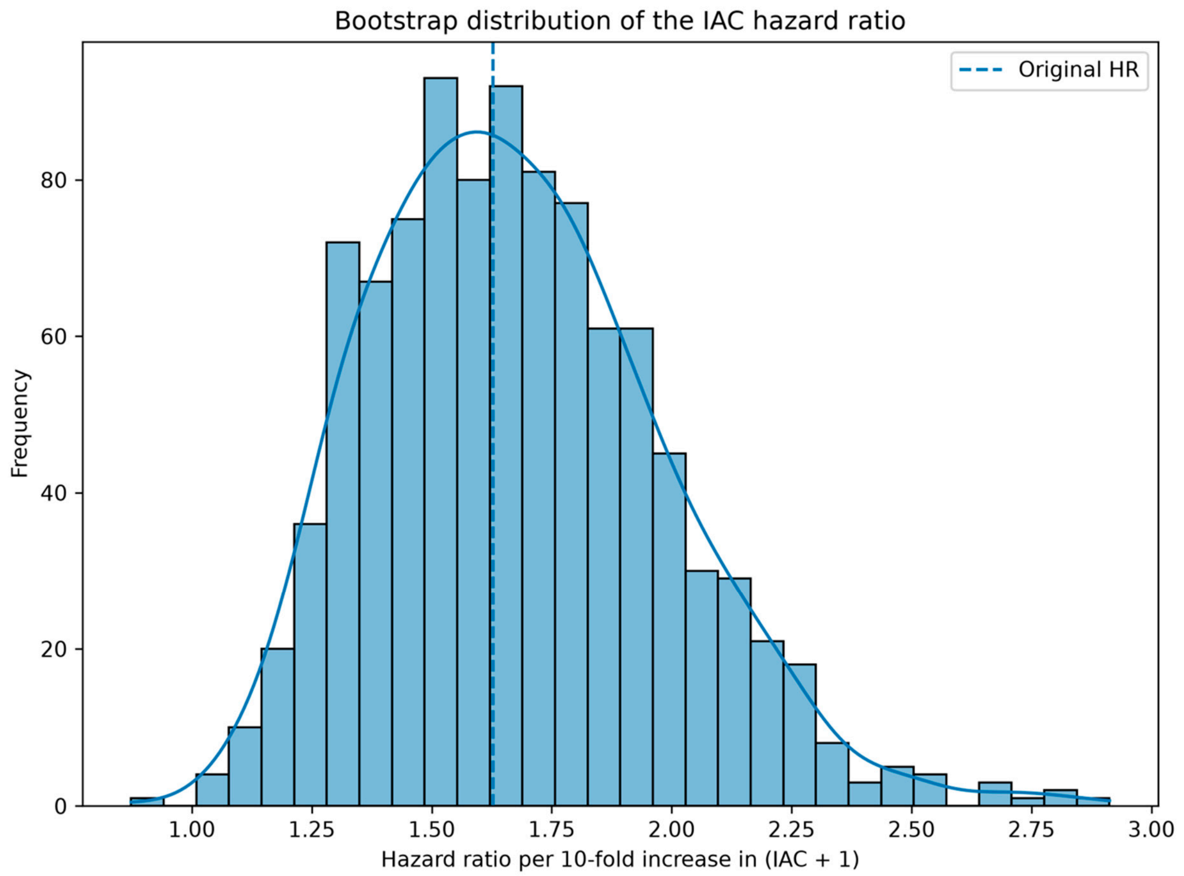

Supplementary Figure S1 Bootstrap internal validation of the multivariable Cox proportional hazards model for recurrent ischemic cerebral events.

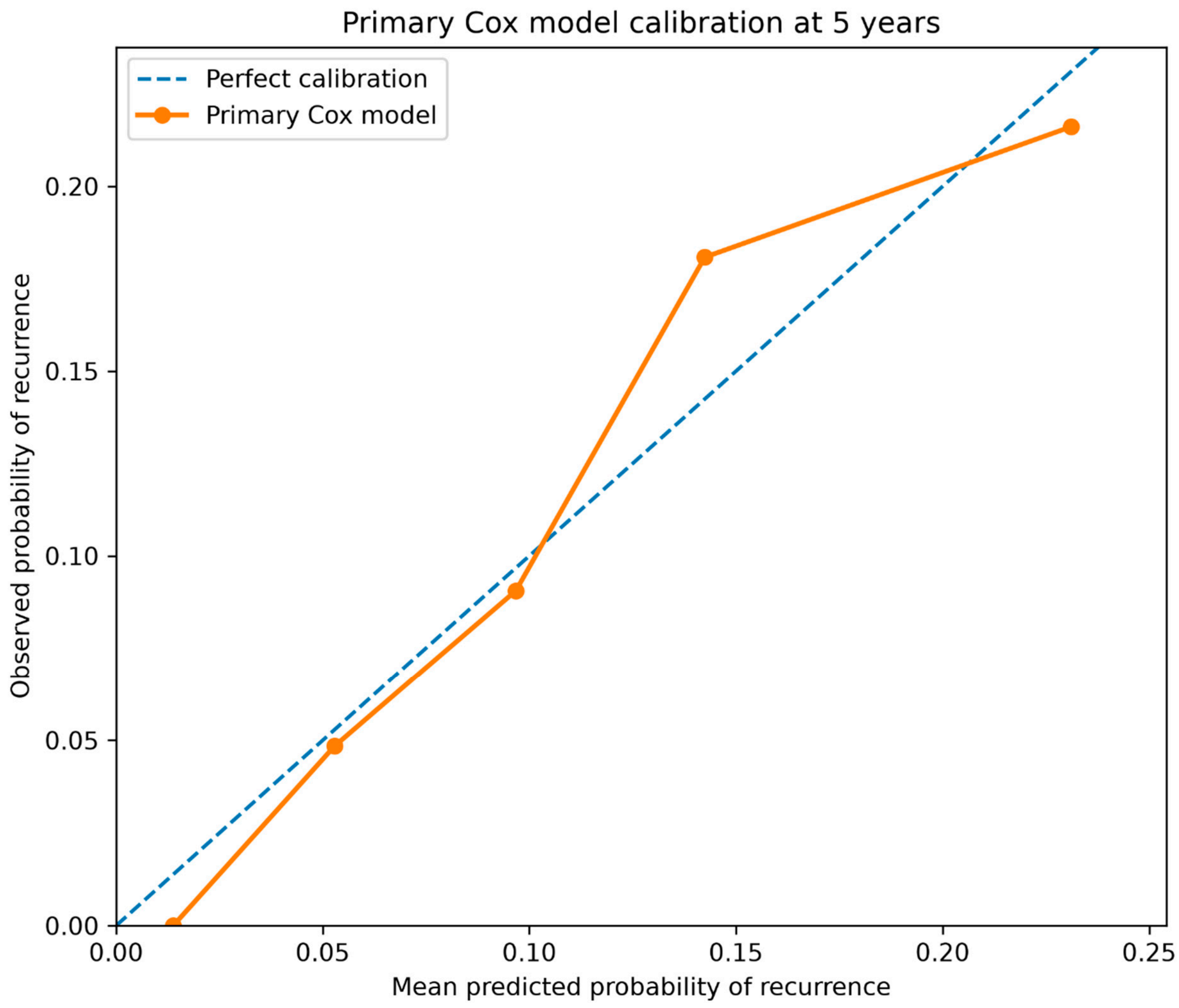

Supplementary Figure S2 Calibration of the primary Cox proportional hazards model at 5 years. Predicted probabilities of recurrent cerebral events were compared with Kaplan–Meier-estimated observed probabilities across five approximately equal-sized groups according to predicted risk. The diagonal line represents perfect calibration.
